# The Emergence of Sequential Buckling in Reconfigurable Hexagonal Networks Embedded into Soft Matrix

**DOI:** 10.3390/ma14082038

**Published:** 2021-04-18

**Authors:** Pavel I. Galich, Aliya Sharipova, Slava Slesarenko

**Affiliations:** 1 Department of Aerospace Engineering, Technion—Israel Institute of Technology, Haifa 32000, Israel; galich.pi@technion.ac.il; 2 Institute of Strength Physics and Materials Science, SB RAS, 634055 Tomsk, Russia; aliya.f.sharipova@gmail.com; 3 Faculty of Mathematics and Mechanics, Saint Petersburg State University, 198504 Saint Petersburg, Russia

**Keywords:** mechanical metamaterials, buckling, sequential buckling, instabilities, elastic wave propagation, reconfiguration

## Abstract

The extreme and unconventional properties of mechanical metamaterials originate in their sophisticated internal architectures. Traditionally, the architecture of mechanical metamaterials is decided on in the design stage and cannot be altered after fabrication. However, the phenomenon of elastic instability, usually accompanied by a reconfiguration in periodic lattices, can be harnessed to alter their mechanical properties. Here, we study the behavior of mechanical metamaterials consisting of hexagonal networks embedded into a soft matrix. Using finite element analysis, we reveal that under specific conditions, such metamaterials can undergo sequential buckling at two different strain levels. While the first reconfiguration keeps the periodicity of the metamaterial intact, the secondary buckling is accompanied by the change in the global periodicity and formation of a new periodic unit cell. We reveal that the critical strains for the first and the second buckling depend on the metamaterial geometry and the ratio between elastic moduli. Moreover, we demonstrate that the buckling behavior can be further controlled by the placement of the rigid circular inclusions in the rotation centers of order 6. The observed sequential buckling in bulk metamaterials can provide additional routes to program their mechanical behavior and control the propagation of elastic waves.

## 1. Introduction

Cellular lattice structures assembled from repeating cells are widely known for their superior combination of low weight and high mechanical properties [1,2,3]. In general, the mechanical performance of lattices is defined by the geometry of the unit cells [4,5,6,7], and rational design of the internal architecture enables the engineering of lattices with enhanced stiffness [8,9], controlled anisotropy of mechanical properties [10,11], or lattices that are tolerant to partial failure [12,13]. Unsurprisingly, many mechanical metamaterials can be considered as successors of cellular materials [14,15]. Indeed, the unconventional properties of mechanical [16,17,18], elastic [19,20,21,22,23], and acoustic metamaterials [24,25] originate in their intrinsic periodicity, along with the rational design of unit cells.

Simultaneously, such intrinsic periodicity can give rise to the elastic instability phenomena often observed in cellular materials and mechanical metamaterials [26]. While for engineering materials loss of stability is usually unwanted, on par with failure or delamination, for functional metamaterials, loss of stability can be frequently harnessed to control their unconventional properties [26,27,28,29,30,31]. The elastic instabilities were used to adjust the stiffness or auxeticity of structured materials [25,32], open and close elastic bandgaps [33], and realize other unusual wave phenomena [34]. Adding a soft deformable matrix into the design facilitates extra coupling between stiff components, enabling more involved buckling behavior accompanied by the formation of various instability-driven patterns [35,36].

The classical and perhaps the simplest examples of such systems in 2D and 3D are periodic layered and fiber composites, respectively. Under uniaxial compression, such composites can undergo elastic loss of stability at a specific value of critical strain defined mainly by the composite geometry and the elastic modulus contrast [37,38,39,40]. In layered and fiber composites, instabilities may develop at different wavelengths ranging from the size of a typical heterogeneity to the size of an entire specimen [41,42]. The onset of macroscopic (or long-wave) instabilities, characterized by a wavelength significantly larger than a characteristic microstructure size, can be predicted by the loss of ellipticity analysis with the homogenized tensor of elastic moduli [43,44,45] that in certain cases can be obtained via micromechanics-based homogenization [46,47,48]. Analysis of microscopic instabilities with wavelengths comparable to the period of a structure requires the application of more involving techniques, such as Bloch–Floquet analysis [42,49]. Surprisingly, the Bloch–Floquet method helped to demonstrate that the secondary loss of stability may occur in bilayer structures under some specific conditions [50]. However, to the best of our knowledge, a realization of such sequential buckling was reported only in the stiff films attached to the soft substrates [51,52,53] or in multilayered structures embedded into the soft media [54,55]. Note that, here, we do not consider cases when the sequential loss of stability occurs due to the change in the metamaterial topology because of contact between elements [56].

Recently, Gao et al. [57] showed that metamaterials consisting of stiff hexagonal networks embedded into a soft matrix undergo loss of stability under biaxial compression. Similar to layered or fiber composites, the instability-induced patterns can be classified as Type 1 or Type 2, corresponding to microscopic and macroscopic instabilities, respectively. In the post-buckling regime, the amplitude of instability-induced patterns increases with an increase in the applied strain, while the overall structure remains the same. It has been shown that such instability-induced transformations can be harnessed to open elastic bandgaps with strain-controlled characteristics. At the same time, the maximal level of applied strain for equibiaxial compression does not exceed 10% for Type 1 and 16% for Type 2 modes. One can notice that in the post-buckling regime, the dispersion curves for metamaterials with Type 1 buckling [57] have values close to zero for the specific wavevectors at the contour of the irreducible Brillouin zone (IBZ). In layered composites, such behavior foreshadows the oncoming loss of stability [34]. An instability occurs when a zero eigenvalue is found for a non-trivial wavevector. In other words, instabilities are associated with zero modes other than rigid body translations and rotations. Zero modes have been observed in Kagome lattices [58] and origami structures [59] and have even been harnessed for elastic cloaking [60].

In the current manuscript, we will explore the buckling phenomenon and demonstrate that stiff hexagonal networks can undergo secondary loss of stability for some specific geometries and material constants. Moreover, we will demonstrate how critical strains for the primary and secondary buckling can be controlled using stiff inclusions placed in the rotation centers of order 6.

## 2. Materials and Methods

**Figure 1:**
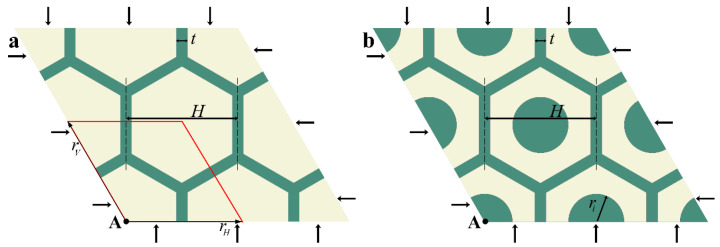
Hexagonal networks embedded into a soft deformable matrix without (**a**) and with (**b**) central inclusions. A primitive unit cell is shown in red color. Lattice vectors $r_{H}$ and $r_{V}$ couple the points on the opposite boundaries of the primitive unit cell.

Figure 1a shows a periodic hexagonal network embedded into a soft deformable matrix. The strut thickness t and the width of a hexagonal element H univocally define the geometry of a metamaterial, while the additional parameter $r_{i}$ is necessary for a metamaterial with central inclusions (Figure 1b). The struts and matrix were considered to be hyperelastic materials with the neo-Hookean strain energy density function

$$W=0.5\mu \left(I_{1}-3\right)-\mu \ln\left(J\right)+0.5\lambda {\left(\ln\left(J\right)\right)}^{2},$$

where $I_{1}$ and J are the first and third invariants of the right Cauchy–Green deformation tensor, and μ and λ are Lame constants that can be expressed through Young’s modulus E  and Poisson’s ratio ν as $\mu =\frac{E}{2\left(1+\nu \right)}$ and $\lambda =\frac{E\nu }{\left(1+\nu \right)\left(1-2\nu \right)}$, respectively. Subscripts N and M stand for network and matrix, respectively. The Poisson ratios of both materials were assumed to be equal, namely, $\nu_{M}=\nu_{N}=0.3$, while $E_{N}/E_{M}>1$. The materials had the same densities $\rho_{N}=\rho_{M}$.

### 2.1. Search for Sequential Instabilities

To facilitate a search for the multiple onsets of instability and analyze the propagation of elastic waves, we used finite element (FE) analysis in COMSOL 5.4. The employment of the FE method for computation of dispersion relations in periodic materials relies on the approach developed in [61,62]. The following multistep procedure, similar to [50], was used to study the reconfiguration of metamaterials undergoing sequential instabilities.

- Search for the first onset of instabilities
  1. We identified the primitive unit cell in the undeformed state (Figure 1a).
  2. We subjected the selected unit cell to equibiaxial compression under plane strain conditions by applying the following periodic boundary conditions:

$$\{\begin{array}{c} u_{right}=u_{left}-\varepsilon H^{h}\\ v_{right}=v_{left}\\ \begin{array}{c} u_{top}=u_{bottom}-\varepsilon H^{v}\cos\frac{\pi }{3}\\ v_{top}=v_{bottom}-\varepsilon H^{v}\sin\frac{\pi }{3}\\ u_{A}=v_{A}=0 \end{array} \end{array}$$

where u and v  are horizontal and vertical displacements, respectively, $H^{h}\;$ and $H^{v}$ are horizontal and vertical periods ($H^{h}=H^{v}=H$ for an initial state), and ε is the applied strain (positive for compression).

3. For the obtained deformed state, we performed a sweep along the perimeter of the IBZ computing dispersion relations ω(k), where ω is the eigenfrequency for the corresponding wavevector k at the IBZ contour [63]. To this end, Bloch–Floquet conditions were superimposed on the finitely deformed metamaterials using the following equations on the primitive unit cell boundaries:

$$\{\begin{array}{c} u_{right}=u_{left}e^{-ikr_{H}}\\ v_{right}=v_{left}e^{-ikr_{H}}\\ \begin{array}{c} u_{top}=u_{bottom}e^{-ikr_{V}}\\ v_{top}=v_{bottom}e^{-ikr_{V}} \end{array} \end{array}$$

where $r_{H}$ and $r_{V}$ are the vectors connecting matching points on horizontal and vertical boundaries of the cell. These equations were implemented in COMSOL using integrated Bloch–Floquet conditions.

4. If ω(k)>0 for all wavevectors k except for the trivial one k=(0,0), then the material remains stable at the specified strain level ε.
5. We repeated steps 2–4, gradually increasing the applied strain ε by 0.01% until the non-trivial $k_{cr}^{1}$ with $\omega \left(k_{cr}^{1}\right)=0$ was found. The determined $\varepsilon_{cr}^{1}=\varepsilon$ and $k_{cr}^{1}$ are the critical strain and critical eigenmode, respectively, corresponding to the *first* or *primary* onset of instabilities.
6. For verification of the buckling mode, we also performed linear buckling analysis [57] to compare critical strains and buckling modes found using these two methods.

B. Subsequent buckling

7. We used the previously found buckling modes as an initial imperfection to guide a reconfiguration of the metamaterial after reaching the buckling strain. If buckling was accompanied by the periodicity change, we increased the unit cell using Floquet continuation. The amplitude of the superimposed imperfection was chosen to be 1/1000 of the stiff layer thickness. The imperfections were superimposed in COMSOL with the help of the Deformed Geometry (dg) interface.
8. We continuously increased the applied strain ε beyond the onset of the first buckling strain and observed the instability-driven transformation of the structure with a gradually increasing buckling amplitude.
9. When necessary, we defined a new primitive cell and updated the IBZ to perform procedure A again, searching for the second onset of instability.

Theoretically, this procedure can be repeated indefinitely to search for sequential onsets of instabilities.

### 2.2. Wave Propagation Analysis

The propagation of the plane elastic waves through the metamaterial was studied using a similar approach due to intricate relations between elastic waves and elastic instabilities [42,63]. Bloch–Floquet boundary conditions (step A3) were superimposed on the finitely deformed metamaterial, and the corresponding eigenproblem was solved for wavevectors k  at the IBZ contour [57].

## 3. Results

### 3.1. Hexagonal Networks Embedded into Soft Matrix

**Figure 2:**
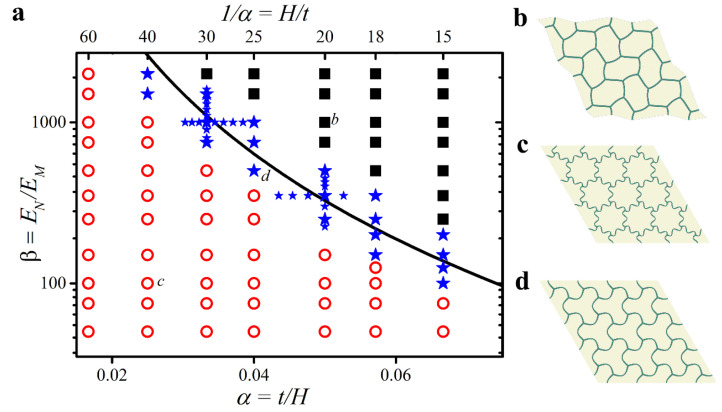
(**a**) Buckling modes as a function of geometrical α and material β parameters. (**b**) Type 2 global mode denoted by solid black squares. (**c**) Type 1 local mode denoted by empty red circles. (**d**) Type 1.5 buckling mode considered in this study denoted by blue stars. The solid continuous line corresponds to the analytical estimation of the transition zone between Type 1 and Type 2 buckling modes, i.e., Equation (1).

First, we recall the classification of two types of buckling modes that can be observed in hexagonal networks embedded into the soft deformable matrix [57]. Similar to the micro-instability and macro-instability, we can distinguish Type 1 buckling with local wrinkling patterns (Figure 2c) and Type 2 buckling accompanied by the formation of a global alternating pattern (Figure 2b). For clarity, we define Type 1 buckling as a case when the metamaterial periodicity remains unaltered, while Type 2 is defined as a case when a new primitive unit cell must be constructed.

Figure 2a shows the map of the eigenmodes as a function of the thickness to the cell ratio α=t/H and the elastic modulus contrast $\beta =E_{N}/E_{M}$. As it was already established for a large enough α and β, Type 2 buckling is realized, while for relatively small values, the metamaterial buckles with the formation of a wrinkling pattern. In general, Type 2 buckling occurs when parameter $\gamma =\alpha \beta^{\frac{1}{3}}>\gamma_{cr}$, defined as

(1) $$\gamma_{cr}=2.17{\left(\frac{3-4\nu }{{\left(1-\nu \right)}^{2}}\right)}^{\frac{1}{3}}$$

Note that this analytical estimation (obtained previously in [57]) is shown here for the specific case of linear elastic constituents with equal Poisson’s ratios $\nu_{M}=\nu_{N}=\nu$. The continuous solid line in Figure 2a corresponds to Equation (1), and it should separate (α, β) pairs associated with Type 1 and Type 2 buckling. However, multiple simulations for various (α, β) pairs reveal that this expression underestimates the threshold value of $\gamma_{cr}$, and microscopic buckling is observed even for $\gamma ≳\gamma_{cr}$ (see the blue stars above the black curve). This slight discrepancy is partially caused by the fact that in Equation (1), the struts of hexagonal networks are treated as Euler–Bernoulli beams. We observed that with an increase in the elastic modulus contrast, the critical value of γ observed in the simulations tends to the critical value defined by Equation (1); however, this is beyond the scope of the current manuscript. While for $\gamma \ll \gamma_{cr}$ metamaterials undergo buckling with the formation of fine wrinkling patterns, here, we focus on the case $\gamma \approx \gamma_{cr}$ associated with Type 1 buckling in which a pattern that accommodates half of the critical wavelength can be observed (Figure 2d). (α, β) combinations corresponding to the formation of this specific pattern are shown by the blue star symbol in Figure 2a. Such a pattern still satisfies the definition of Type 1 buckling since there are no changes in the periodicity. However, the vicinity of such metamaterials to the configurations with global buckling (Type 2) implies that critical buckling strains associated with Type 1 and Type 2 buckling may be very close to each other, enabling conditions for the subsequent loss of stability. Therefore, we will further focus mainly on the buckling behavior of these intermediate *Type 1.5* metamaterials.

**Figure 3:**
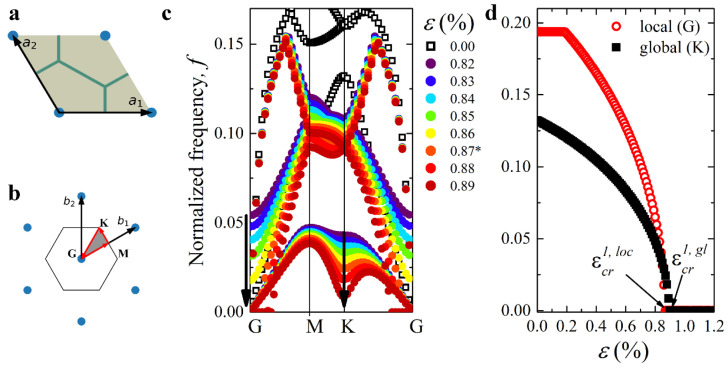
The primitive unit cell (**a**) and its reciprocal lattice (**b**) of the studied structures in the undeformed state. The gray area represents the IBZ, and the red arrows show the path along its contour. (**c**) Evolution of lowest branches of dispersion curves in the vicinity of the primary instability. * denotes the minimal strain (ε=0.87%) for which a non-trivial zero eigenvalue is found at point G. (**d**) Evolution of the eigenfrequencies corresponding to Type 1 and Type 2 modes with an increase in the applied strain. The *Y*-axis represents normalized frequency $f=\frac{\omega H}{2\pi }\sqrt{\frac{\rho_{M}}{\mu_{M}}}$.

**Figure 4:**
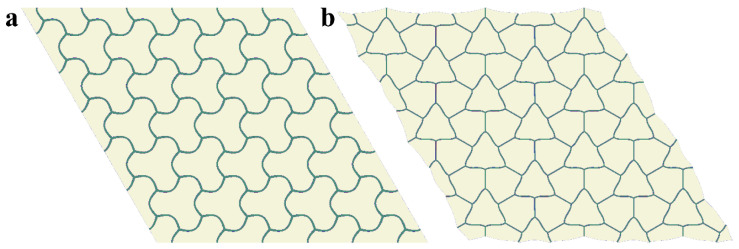
Type 1 (**a**) and Type 2 (**b**) patterns associated with the first onset of buckling.

Figure 3c,d illustrate the behavior of elastic waves in the mechanical metamaterial with α=1/30 and β=1000 in the vicinity of the first onset of elastic instability. In particular, Figure 3c shows the evolution of the lowest branches of dispersion curves observed as the applied strain ε increases. One can see that an increase in the applied strain leads to a decrease in the phase velocity of elastic waves. In physics, this phenomenon is also known as mode “softening” [64]. According to the procedure described above, metamaterial remains stable when ω(k)>0 for all k. Note that for k=(0,0), there is always a trivial solution ω(k)=0, associated with the rigid body movement. At the same time, since Type 1 buckling does not cause the change in periodicity, to find the onset of Type 1 buckling, we track the second lowest shear branch of the dispersion diagram, focusing on a non-trivial solution for k=(0,0). Figure 3d shows how the eigenvalue $\omega^{loc}=\omega \left(G\right)$ computed for vector k=(0,0), corresponding to point **G** of the IBZ contour, decreases with an increase in the applied strain until it reaches $\omega_{cr}^{loc}=0$ for the strain $\varepsilon_{cr}^{1,\;loc}=0.87\%$. This critical strain corresponds to the first onset of the instabilities associated with Type 1 local buckling, and the corresponding buckling mode is shown in Figure 4a. Surprisingly, simultaneously, the eigenfrequency $\omega^{gl}=\omega \left(K\right)$ computed for point **K** of the IBZ contour also noticeably decreases with the increase in ε, especially in the vicinity of $\varepsilon_{cr}^{1,\;loc}$. The pattern shown in Figure 4b corresponds to point **K**, and this pattern has a different periodicity compared to the undeformed state. However, $\omega^{gl}>0$ for $\varepsilon_{cr}^{1,\;loc}$; therefore, Type 1 buckling occurs first. Realization of buckling should lead to the change in the geometry of the unit cell; however, if no perturbations are superimposed on the undeformed state, the numerical methods may fail to detect the buckling mode, allowing us to reach higher strains without any reconfigurations.

**Figure 5:**
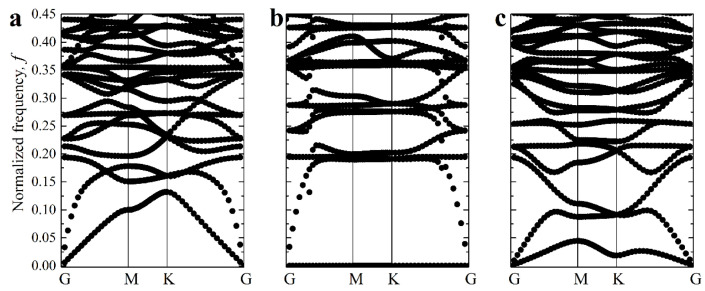
Dispersion curves for metamaterial with α=1/30 and β=1000. (**a**) Undeformed state, (**b**) ε=7% without reconfiguration, (**c**) ε=7% with reconfiguration.

Figure 5a shows the dispersion diagrams for metamaterials in undeformed (ε=0%) and deformed (ε=7%) states. Note that the dispersion curves for the deformed state are obtained for two distinct cases—with (Figure 5c) and without (Figure 5b) reconfiguration, triggered by the first onset of instabilities at $\varepsilon_{cr}^{1,\;loc}=0.87\%$. One can see that without reconfiguration, the dispersion curves continuously move towards the *x*-axis, enabling us to define $\varepsilon_{cr}^{1,\;gl}=0.90\%$ as the lowest strain level, such as $\omega^{gl}=0$ (see Figure 3d). The linear perturbation analysis confirms that this critical value $\varepsilon_{cr}^{1,\;gl}$ and the corresponding eigenmode (Figure 4b) can be observed as the second minimal buckling mode if the enlarged unit cell, consisting of nine primitive unit cells, is considered. However, the second value $\varepsilon_{cr}^{1,\;gl}$ can be reached only because the instability-driven transformation associated with Type 1 buckling was restricted. Figure 5b reveals that if the reconfiguration does not occur, after the onset of local instability in the post-buckling regime, ω(k)=0 for the selected directions k, and, therefore, the phase velocity of the waves propagating through the metamaterial is equal to zero [65]. Zero phase velocity corresponds to the degenerate case, and the existence of zero eigenvalues after the bifurcation hints at inconsistencies in numerical analysis. In contrast, if reconfiguration takes place, zero eigenfrequencies are not observed apart from trivial ones (Figure 5c), and the metamaterial maintains its stability in the updated configuration.

**Figure 6:**
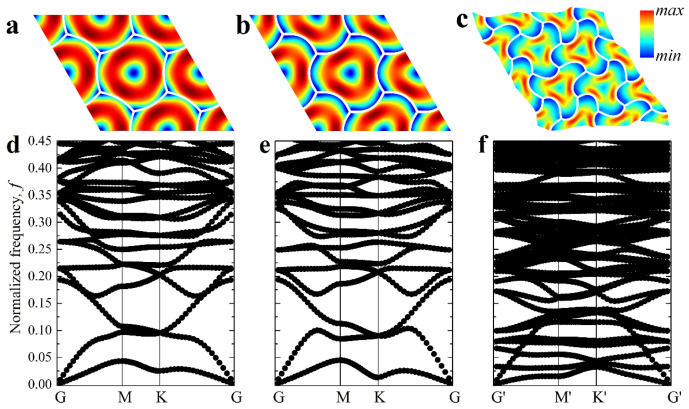
Evolution of the pattern (**a**–**c**) and corresponding dispersion curves (**d**–**f**) after the first and the second buckling for metamaterial with α=1/30 and β=1000. (**a**,**d**) ε=3%, (**b**,**e**) ε=9%, (**c**,**f**) ε=25% (updated IBZ is shown in Figure 7b). Colors denote von Mises stresses (color ranges are not synchronized for (**a**–**c**)).

**Figure 7:**
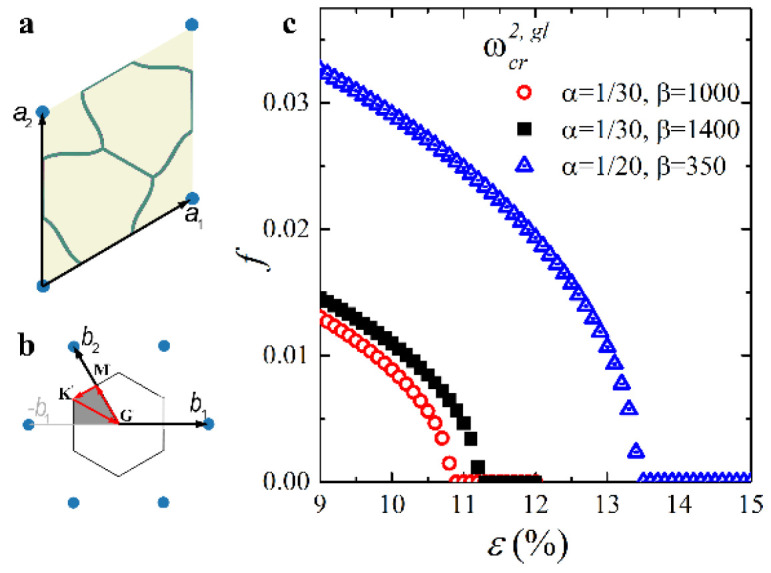
The primitive unit cell (**a**) and its reciprocal lattice (**b**) of the studied structure after secondary buckling. The updated (virtual) IBZ contour is shown in red [66]. (**c**) Evolution of the lowest eigenfrequency at point **K** in the vicinity of primary instability.

**Figure 8:**
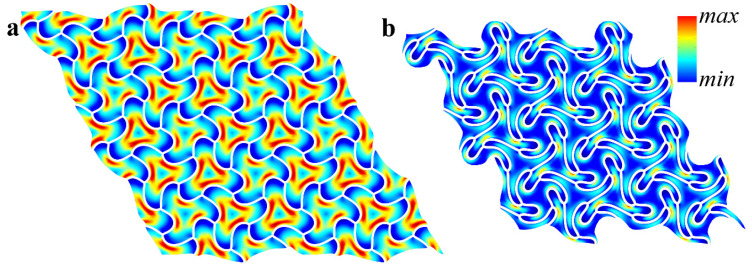
Evolution of the pattern after the second buckling at ε=30% (**a**), ε=50% (**b**). Colors denote von Mises stresses (color ranges are not synchronized for (**a**, **b**)).

Figure 6 shows the pattern evolution after the first buckling. With an increase in the applied strain ε, the amplitude of the pattern gradually increases (compare Figure 6a,b), while the node positions continue to form the right hexagons, proving that the metamaterial keeps its initial periodicity even in the post-buckling regime. However, according to Figure 7c, with a further increase in the applied strain, the value $\;\omega^{G}$ starts to tend to zero again. Eventually, $\omega^{G}=0$ for the value of strain $\varepsilon_{cr}^{2,gl}=10.8\%$ (Figure 7c). Since the zero eigenfrequency is observed for point **K**, the metamaterial undergoes secondary buckling accompanied by global reconfiguration with the formation of the same pattern as for hypothetical global buckling canceled by the first local reconfiguration at $\varepsilon_{cr}^{1,\;loc}=0.87\%$ (Figure 4b). However, since the local patterns have already developed prior to the second onset of the instabilities, the resulting patterns in the post-buckling regime (after secondary buckling) gain a mixed geometry combining the features from both buckling modes (Figure 8). This sequential buckling lowers the plane group of the metamaterial from p6mm in the undeformed state down to p3 (e.g., see [66] for a reference regarding the plane groups). With the further increase in the applied strain ε, the formed pattern continues to evolve. At the same time, one can see that the applied strain does not significantly affect the dispersion curves after the first reconfiguration (Figure 6d,e), and the secondary buckling leads to the noticeable increase in the density of dispersion curves (Figure 6f) due to the more complex geometry of the new primitive cell.

**Figure 9:**
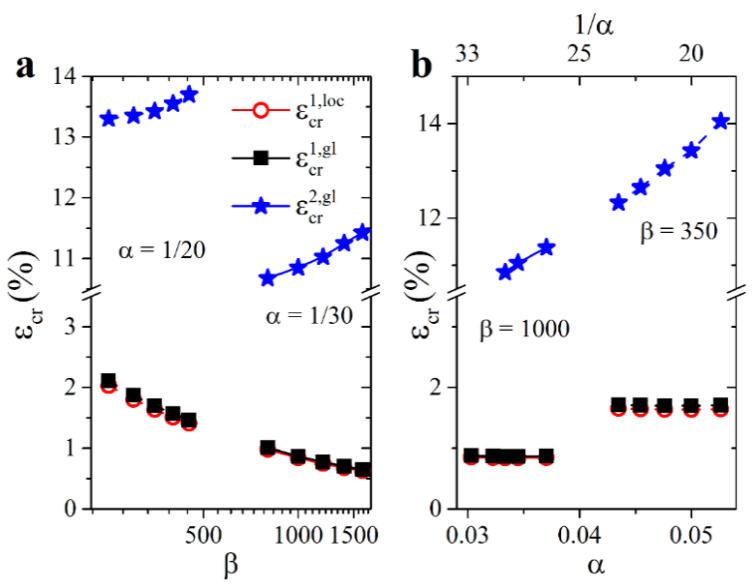
Dependencies of the first and the second buckling strains on material parameter β (**a**) and geometrical parameter α (**b**).

We note that all considered Type 1.5 metamaterials (Figure 2a) undergo the subsequent loss of stability. Therefore, one can conclude that for selected α and β such as $\alpha \beta^{\frac{1}{3}}\approx \gamma_{cr}$, Type 1 local buckling does not cancel the Type 2 instability but barely postpones it. For instance, for the metamaterial with α=0.05 (H/t=20) and β=350, global buckling occurs only after an additional 12% strain ($\varepsilon_{cr}^{2,\;gl}-\varepsilon_{cr}^{1,\;loc}\approx 11.7\%$). Figure 9 shows the values of the critical buckling strains that correspond to the first and secondary onsets of instability for the selected metamaterials denoted by smaller star symbols in Figure 2a. Due to the definition of Type 1.5 metamaterials $\varepsilon_{cr}^{1,\;loc}<\varepsilon_{cr}^{1,gl}$, one may notice that the geometric factor α does not affect the primary buckling in any significant way (Figure 9b). In contrast, an increase in parameter α, for instance, due to the wider struts of the hexagonal network postpones the secondary buckling, making the metamaterial more stable. A more significant effect is observed concerning the material factor β. Figure 9a reveals the opposite dynamics for the onsets of the first and second instabilities. While metamaterials with a higher elastic modulus contrast undergo primary buckling at lower strains, the subsequent secondary buckling is postponed as compared with metamaterials with a lower elastic modulus contrast. Hence, the strain range between two consecutive bucklings is wider for the metamaterials with a higher contrast between the elastic moduli of constituents. For example, for the mechanical metamaterial with α=1/30 and β=800, the width of such a strain range is 9.7% versus 10.8% for a metamaterial with β=1800. In general, since Type 1 buckling induces fewer perturbations in the architecture of mechanical metamaterials, in some practical cases, it may be practical to alter the geometric parameters of the hexagonal networks with the intention to broaden the operation conditions by postponing the global buckling. However, the observed effect is relatively minute and requires a precise selection of α and β; therefore, even slight fabrication imperfections may undermine the desired effect.

### 3.2. Hexagonal Networks with Embedded Inclusions

As the soft matrix in the considered metamaterials couples the deformation of the stiff struts on the unit cell boundaries, the additional modification of the soft matrix seems to be a promising way to tune their stability and control the propagation of elastic waves. Thus, we modify the architecture of the metamaterials by placing a stiff circular inclusion in the rotation center of order 6—the center of the soft hexagon (Figure 1b). It was previously shown, however, that various systems consisting of rigid inclusions embedded into a soft matrix could undergo instability-driven transformations with the formation of wavy patterns or twinning domains for a singular loss of stability [67]. Here, we harness inclusions (without reducing the symmetry group of the metamaterial) as an additional factor, enabling us to control the values of critical buckling strains. We select the elastic constants of the inclusions to match the elastic constants of the stiff network, causing the inclusions to be virtually undeformable.

**Figure 10:**
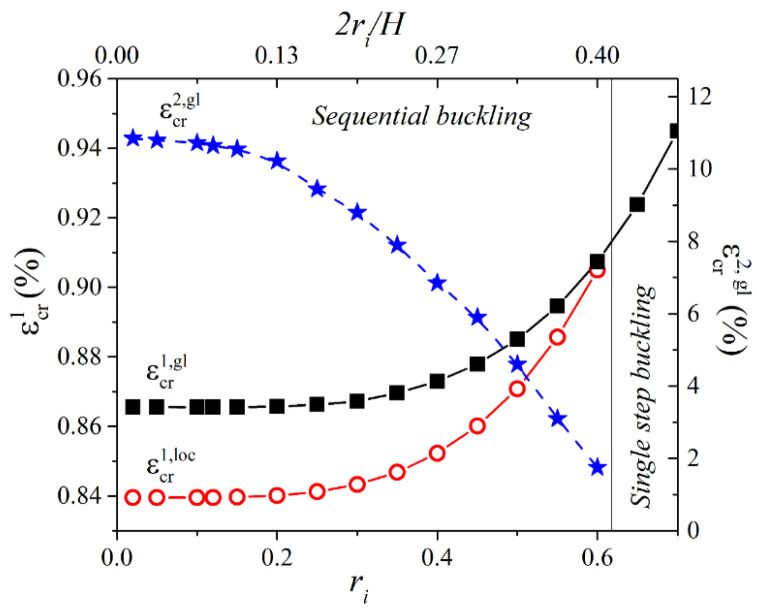
Values of critical strains for the hexagonal networks with embedded circular inclusions.

Figure 10 shows dependencies of the values of critical strains ($\varepsilon_{cr}^{1,\;loc}$, $\varepsilon_{cr}^{1,gl}$, and $\varepsilon_{cr}^{2,gl}$) on the radius of the inclusions $r_{i}$ and dimensionless parameter $\delta =2r_{i}/H$ for metamaterials with α=1/30 and β=1000. One can see that relatively small inclusions do not significantly affect the first onset of instabilities, and the metamaterial still undergoes two subsequent transformations. However, with an increase in the inclusion radius, the difference between the two first buckling modes ($\varepsilon_{cr}^{1,gl}-\varepsilon_{cr}^{1,\;loc}$) starts to decrease until $\varepsilon_{cr}^{1,loc}>\varepsilon_{cr}^{1,gl}$ approximately when δ≈0.4, representing the transition from the configuration with sequential buckling to the configuration with single-step buckling. Simultaneously, the critical strain corresponding to the secondary buckling monotonically decreases with an increase in the inclusion radius. Finally, for δ≈0.4, which corresponds to the transition between modes, we have $\varepsilon_{cr}^{1,\;loc}=\varepsilon_{cr}^{1,gl}=\varepsilon_{cr}^{2,gl}$. Hence, the addition of the central inclusions proves to be an efficient strategy to control buckling behavior in metamaterials consisting of hexagonal networks embedded into a soft deformable matrix.

**Figure 11:**
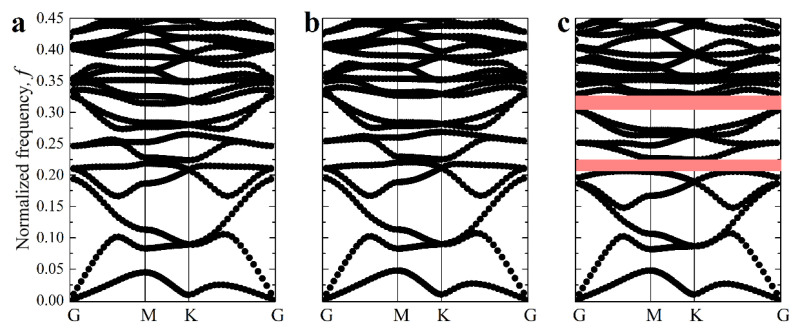
Dispersion curves for the deformed composite with α=1/30, β=1000, δ=0.1 after primary buckling and ε=10% for metamaterials with no inclusions (**a**), $\rho_{I}/\rho_{M}=1$ (**b**), $\rho_{I}/\rho_{M}=8$ (**c**). The red rectangles denote complete bandgaps for in-plane waves.

While embedded inclusions are shown to affect the buckling behavior, they can also alter the propagation of the elastic waves in metamaterials, especially if the density contrast between the soft matrix and inclusions can be realized. Figure 11 shows dispersion curves for the selected metamaterial with α=1/30, β=1000, δ=0.1  deformed up to 10%. One can see that while the dispersion curves for the metamaterials without (Figure 11a) and with (Figure 11b,c) inclusions are very similar, the additional density contrast between embedded inclusions may facilitate the opening of the elastic bandgaps (Figure 11c). Since the localized masses can be employed to design locally resonant elastic metamaterials, the change in the architecture caused by the elastic instabilities in the hexagonal networks could be potentially harnessed to control bandgaps.

## 4. Conclusions

We revisited the mechanical behavior of the stiff hexagonal networks embedded into the soft matrix, primarily focusing on the phenomenon of elastic loss of stability. Previously, two different types of instability-driven transformations were distinguished depending on the periodicity of the emerged patterns. In this paper, we revealed that mechanical metamaterials consisting of stiff hexagonal networks embedded into the soft matrix could undergo sequential buckling for the specific combinations of geometric and material parameters. Under equibiaxial compression, these metamaterials first undergo local buckling, keeping their periodicity in the post-buckling regime. However, with the further increase in the applied strain, a secondary loss of stability accompanied by the formation of new patterns can be triggered. We studied how geometrical and material factors affect the buckling behavior. In particular, we demonstrated that the elastic modulus contrast affects the values of the critical strain for primary and secondary losses of stability in opposite ways: while the metamaterial becomes less stable with an increase in contrast between elastic moduli, the critical strain corresponding to the secondary buckling increases.

The method to search for the sequential instabilities using COMSOL was described. Moreover, we highlighted the importance of the minute perturbations in the undeformed states for numerical analysis to avoid degenerate dispersion curves with zero phase velocities. This analysis helped us to show that even though initially two eigenvalues associated with local and global buckling might be close to each other, the primary loss of stability can significantly postpone the secondary buckling, accompanied by the global reconfiguration. We demonstrated that the placement of additional stiff inclusions in the rotation centers of order 6 can be employed to control the buckling behavior and propagation of elastic waves. In conclusion, we note that the experimental verification of the described phenomena can be a challenging task due to the relatively narrow region in the design space for which sequential buckling is observed, and imperfections associated with the manufacturing process [68,69]. Nevertheless, we believe that these results are useful for engineering other bulk systems with sequential buckling.
